# Aging Resistance of Silica Fume/Styrene-Butadiene-Styrene Composite-Modified Asphalt

**DOI:** 10.3390/ma14216536

**Published:** 2021-10-30

**Authors:** Jingrong Zhu, Wenyuan Xu

**Affiliations:** School of Civil Engineering, Northeast Forestry University, Harbin 150040, China; zhujingrong@nefu.edu.cn

**Keywords:** silica fume, composite-modified asphalt, rheological properties, aging resistance, aging mechanism

## Abstract

The influences of silica fume content and aging on the rheological properties of silica fume/styrene-butadiene-styrene composite-modified asphalts were investigated via rolling thin-film oven test simulations. The asphalts rheological properties before and after aging were measured using three-major-indices, dynamic shear rheology, and bending beam rheometer tests. Fourier transform infrared spectroscopy was used to examine the changes in the functional groups of the asphalt. The silica fume did not chemically react with the modified asphalt, and its original structure was maintained. The aging resistance improved significantly after adding the silica fume. At 6% silica fume content, the relaxation of the asphalt was the highest, indicating that the asphalt had the best low-temperature crack resistance at this mixing proportion. Furthermore, the carbonyl index value of this sample exhibited the smallest increment among all of the samples, and this asphalt sample had the strongest short-term aging resistance. Thus, the optimum silica fume content in the composite-modified asphalt was determined to be 6%. This information may be used to fabricate an asphalt mixture that can improve the service life and aging resistance of pavements.

## 1. Introduction

The problem of the insufficient durability of asphalt pavement due to aging has always been the focus of road researchers both locally and internationally. Aging means that the asphalt is oxidized, decomposed, and aggregated under the influence of heat, oxygen, ultraviolet radiation, and water. Its internal molecular structure and chemical composition change during transportation, construction, and pavement use after it is produced, thus resulting in the deterioration of its properties [1,2,3].

The aging of asphalt pavement will result in a series of deformities such as cracks and subsidence, which will create a vicious cycle under the action of rainwater and driving that accelerate the aging of asphalt and cause further aggravation of pavement deformities. Therefore, it is necessary to add modifiers to asphalt to improve the durability and aging resistance so as to prolong the service life of pavement [4,5].

Modifiers are mainly divided into two categories: organic and inorganic. Among organic modifiers, styrene-butadiene-styrene (SBS) is the most commonly used modifier. SBS-modified asphalt exhibits excellent road performance, as well as adequate high-and low-temperature properties that fulfil the requirements of pavements [6,7,8,9,10]. However, due to the great difference in composition and structure between the SBS modifier and asphalt, it is difficult to form a stable system during blending and they are prone to phase separation during thermal storage [11]. Moreover, SBS can be degraded during thermal oxygen aging which will further accelerate the aging of the asphalt, resulting in the decline o+f asphalt durability [12]. Therefore, it is necessary to add stabilizing inorganic materials to the asphalt to suppress these negative properties. Silica fume is an inorganic material, which is characterized by its abundance, low cost, high specific surface area, good thermal stability, and strong adsorption capacity. Asphalt that is modified by silica fume generally exhibits improved pavement properties, including its rutting resistance, durability, and aging resistance [13,14,15,16].

Recent research has demonstrated that the performance of base asphalt can be improved to a greater extent by simultaneously adding both organic and inorganic modifiers compared to adding only one type [17]. Feng et al. [8] studied the high-temperature rheological properties of silica fume/SBS composite-modified asphalt and demonstrated that the addition of silica fume increased the plural shear modulus and rutting resistance factor of the composite-modified asphalt, while also improving its high-temperature rheological properties. Li et al. [18] found that Nano-CaCO_3,_ nano-ZnO, and SBR can be evenly dispersed in the composite-modified asphalt meaning that the structural stability of the modified asphalt can be improved.

Only when durable materials are selected for construction, can the quality of buildings be improved, service life be extended, and maintenance costs be reduced. Recently, people are attaching greater importance to the durability of materials [19,20]. Ye et al. [21] analyzed the aging properties and viscosity-temperature characteristics of modified asphalt that contained 5% nano-SiO_2_. Their results showed that nano-SiO_2_ effectively improved the viscosity and durability of asphalt.

The published studies in the literature mainly focused on the rheological properties of silica fume modified asphalt, however, only a few articles on its anti-aging properties were evaluated with the data of three-major-indices tests. We share the same viewpoint with Huang [22] that such evaluation has certain limitations and a more perfect evaluation system should be used. In addition, it is indispensable to provide an insight into the microscopic variations of aging materials. Zhang [23] and Lesueur [24] have conducted in-depth research on this; only with a clear understanding of the aging mechanisms can we find the more targeted method to improve the durability of asphalt. Despite this, research on the aging mechanisms of silica fume/SBS composite-modified asphalt is limited.

Based on the excellent performance of silica fume/SBS composite-modified asphalt, this study investigates the short-term thermal-oxygen aging of composite-modified asphalt with different silicon powder contents. By analyzing the rheological properties and the functional group changes before and after aging, the influence of the silicon powder content and aging on the properties of the composite-modified asphalt and its aging mechanism were examined. The results of this study will be used to further promote the application of inorganic materials and organic polymers in composite-modified asphalt for pavement construction projects.

## 2. Materials and Methods

### 2.1. Materials

The 4% SBS-modified asphalt was prepared by mixing AH #90 base asphalt (Panjin, China) with an YH-791H SBS modifier (Type 1301). The properties of the resulting asphalt are listed in Table 1.

The micro-silica fume was obtained from the American Trade Industrial Development Co., Ltd. (Anshan, China), and its properties are listed in Table 2.

### 2.2. Preparation Methods

#### 2.2.1. Asphalt Preparation

According to the research of [25,26], when the content of SBS modifier is 4%, it can be miscible with asphalt to form the most stable network structure. As such, the percentage of the SBS modifier that was added to asphalt was chosen to be 4%.

A review of past literature [4,27] revealed that the low temperature performance of asphalt will greatly reduce when the silica fume content exceeds a certain level. After comprehensive consideration of previous research [28,29], the silica fume content with mass fractions of 2%, 4%, 6%, and 8% of asphalt was selected in this study.

After heating the SBS-modified asphalt to a molten state, different percentages of silica fume were added. A FLUKOAF25 high-speed shear stirring machine was used to shear at 165 °C and 5000 rpm for 60 min, followed by stirring at a rate of 300 rpm for 5 min to remove the small bubbles that were remaining in the asphalt after undergoing high-speed shearing. According to the specifications of the *JTG E20—2011 T0610-2011*, the test samples were aged using the rolling thin-film oven (Changji Geological Instrument CO., LTD, Shanghai, China) test at 163 °C for 85 min, with an air flow rate of 4000 mL/min, and then prepared for subsequent testing.

In this study, three groups of parallel samples were conducted for each test to ensure the accuracy of data.

#### 2.2.2. Three-Major-Indices Tests

According to the specifications of the *JTG E20—2011*, the three-major-indices tests of the asphalt were first conducted.

#### 2.2.3. Dynamic Shear Rheological (DSR) Test

Through the DSR test, the variations in the rutting factor (G*/sin δ) and phase angle (δ) were analyzed, which were used to evaluate the high-temperature deformation resistance of asphalt, as well as the indices of the high-temperature rheological properties of the composite asphalt. Based on the test standard of *AASHTO 315-09*, this experiment used a MCR302 DSR (Anton Paar, México, Germany), and a constant 12% strain to conduct the temperature sweep test. The loading frequency was 10 rad/s, the initial temperature was 58 °C, the temperature-rise range was 6 °C, and the highest temperature was 76 °C. The sample size was 25 mm and the spacing was 1 mm [30].

#### 2.2.4. Bending-Beam Rheometer (BBR) Test

In a low-temperature environment, asphalt changes from a viscoelastic to a brittle state. Therefore, improving the low-temperature performance of asphalt materials is a critical issue. According to *AASHTO 313-09* test standard, the low-temperature CANNON TE-bending-beam rheometer (BBR) (State College, PA, USA) was used to perform the loading test. The loading test was carried out at −12, −18, and −24 °C for 240 s, and measurements were obtained at 8, 15, 30, 60, 120, and 240 s. The creep stiffness modulus (S) and the creep rate (m) at 60s were selected for the subsequent analysis.

#### 2.2.5. Fourier Transform Infrared (FTIR) Test

This experiment was based on the test standard of *DB14/T 2320-2021*. A PerkinElmer Spectrum 400 series FTIR spectrometer (PerkinElmer, Shanghai, China) was used to dissolve the asphalt samples in dichloromethane to make a 10% solution. The KBr wafer was scanned as a blank background before testing, and one drop of the solution was added to the KBr wafer. The asphalt sample was analyzed using the above-mentioned infrared spectrometer. The scanning range and number of scans were 4000–400 cm^−1^ and 120, respectively. The infrared spectrum was analyzed using OMNIC processing software (Version 8.2, Thermo Nicolet, Madison, WI, USA) to characterize the structures of the different asphalt samples and their functional groups before and after aging.

## 3. Results and Discussion

### 3.1. Basic Physical Performance Indicators

Following the preparation methods that were mentioned previously, 2%, 4%, 6%, and 8% amounts of silica fume were mixed into the asphalt samples, and the asphalt was aged. According to the specification of *JTG F40-2004* for SBS modified asphalt, the penetration at 25 °C should be more than 60 mm, the ductility at 5 °C should not be less than 30 cm and the softening point is not less than 55 °C. After short-term aging (RTFOT), the penetration ratio at 25 °C was not less than 60 and the ductility at 5 °C was not less than 20 cm.

The results of the three-major-indices tests are shown in Table 3 and Table 4.

**Figure 1:**
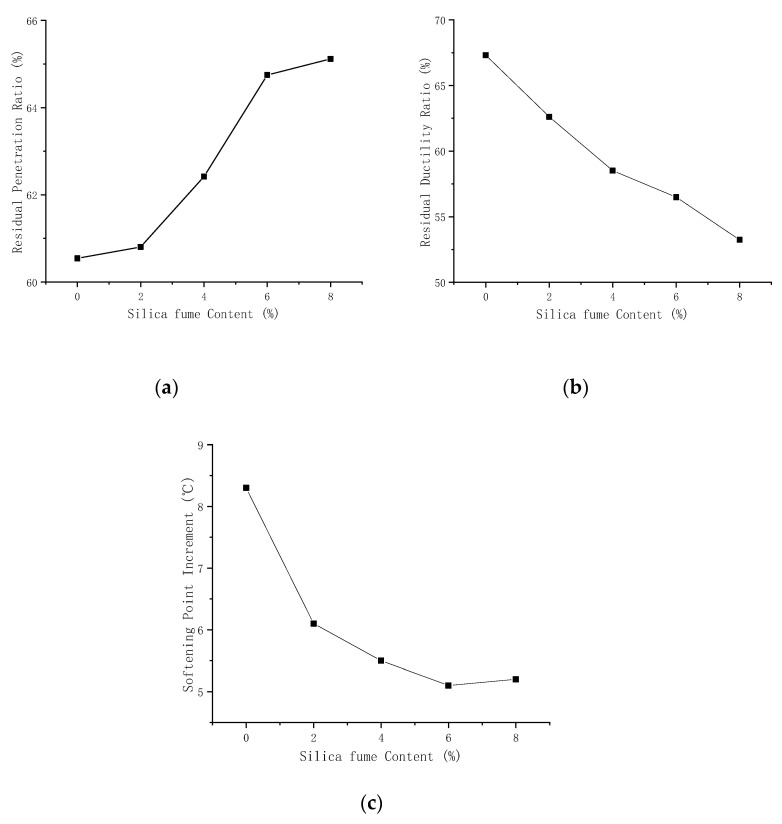
Plots of the temperature and the (**a**) residual penetration ratio, (**b**) residual ductility ratio, and (**c**) softening point increment.

As shown in Table 3 and Table 4 and Figure 1, The test data of penetration and softening point of the asphalt with each silica fume content met the standard.

When the silica fume content was increased, the penetration of the asphalt decreased, while the penetration retention rate gradually increased. The penetration retention rate changed most significantly when the silica fume content was increased from 4% to 6%, but when the content exceeded 6%, the penetration retention rate became constant. Due to its characteristic of a large specific surface area, the silica fume can absorb light components from the asphalt and thus improve the consistency [28,31]. However, as mentioned by [27,32], its improvement capability was not obvious when the silica fume content exceeded a certain level.

The softening point of the composite-modified asphalt increased with the increase of silica fume content. The addition of silica fume caused the asphalt to swell and the cementitious composition gradually increased, so the softening point increased macroscopically [33]. We also found that the softening point after aging was higher than that before aging, and the increment decreased gradually with the increase of silicon powder content. This conclusion can also be confirmed by [34], where their findings suggest that the micro pore structure of silica fume is equivalent to that of microcapillaries. This has a capillary action that can enhance the interfacial forces between the silica fume and the asphalt. This inhibits the redox reaction between the asphalt and oxygen to a certain extent and thereby plays an anti-aging role. However, it is basically consistent with the conclusion of penetration that is discussed above, that when the content of silicon fume is too high, it will have an adverse impact on the modification effect.

The ductility and residual ductility ratio both exhibited a gradual decrease after the addition of the silica fume. This indicates that the ability of the asphalt to withstand plastic deformation decreases with the addition of silica fume. Zheng’s [28] study shows that this may be due to the unstable cross-linking structure that is formed by the silica fume and SBS modified asphalt at lower temperatures. With the increase of silica fume content, this unstable structure becomes more unstable and results in an increase in the non-uniformity of the asphalt material, leading to poor rheological properties.

### 3.2. High-Temperature Rheological Properties

According to the test method in 2.2.3 above, the composite-modified asphalt before and after aging, with 2%, 4%, 6%, and 8% silica fume contents, were selected for the DSR tests. The anti-rutting factor G*/sinδ was proposed in SHRP to evaluate the high temperature rutting resistance of asphalt. The larger the value, the better the rutting resistance and the stronger the permanent deformation resistance of asphalt in a high temperature environment. SHRP also specifies that the temperature at G*/sinδ = 1 is the failure temperature of asphalt before aging.

**Figure 2:**
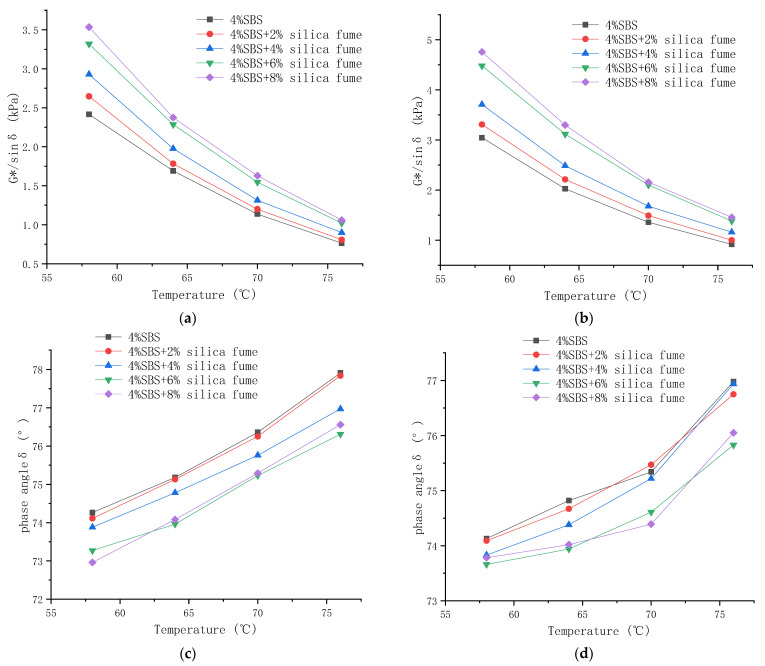
(**a**) Relationship between G*/sin δ and the temperature before aging and (**b**) after aging; (**c**) relationship between the phase angle (δ) and temperature before aging and (**d**) after aging.

The test results are shown in Figure 2.

From Figure 2a,b, it can be seen that the rutting resistance factor of the modified asphalt gradually decreased with an increase in temperature, and its rate of decrease gradually decreased with increasing temperature. At the same temperature, G*/sin δ increased with the addition of silica fume. Upon comparison, it was found that the rutting resistance factor of the aged asphalt was significantly higher than that of the asphalt before aging. The increase in this factor is greater for the composite-modified asphalt than that for the SBS-modified asphalt.

This is consistent with the opinion of [33,35], which show that silica fume can significantly improve the permanent deformation resistance of asphalt. The main reason is that silica fume belongs to a group of inorganic materials with low thermal conductivity. This makes silica fume have good thermal stability when used as modifier to make composite-modified asphalt. It can reduce the thermal conductivity of composite-modified asphalt and improve its high-temperature stability. When the asphalt is aged, the heavy components (asphaltene and gum) in the asphalt increase and the light components (saturated and aromatic components) decrease. The addition of silica fume can enhance the intermolecular force and reduce the flow strength of the composite-modified asphalt. Therefore, silica fume can play a role in improving the anti-aging properties of asphalt in high temperature environments.

As can be seen from Figure 2c, all of the phase angles of the modified asphalt exhibited a tendency to increase with the increase in temperature. This indicates that the viscous components of asphalt increase while the elastic components decrease as the temperature is increased. This is consistent with the fact that asphalt exists mainly in an elastic state at a relatively low temperature and gradually changes into a mainly viscous state as the temperature increases. By comparing the phase angles over the entire experimental temperature range, it was observed that the curve of the phase angle versus the temperature of the asphalt without silica fume was higher than those of the other examined samples. This indicates that the elastic recovery of asphalt can be improved by adding silica fume, and the improvement effects become stronger as the silica fume content is increased.

As shown in Figure 2d, the phase angle of the asphalt after aging was reduced compared with that before aging. This comparison also shows that the range of increase in the phase angle for the composite-modified asphalt after aging was much larger than that of the SBS-modified asphalt. This is because aging transforms the light components in the asphalt into heavy components. Silica fume can absorb the light components and inhibit their volatilization, thereby improving the aging resistance of the asphalt. However, when the amount of silica fume exceeded a certain limit, the agglomeration effect of the nanoparticles increased, and the increase in the modification effects was less evident.

### 3.3. Formulation of the Regression Equation

To further observe the influence of silica fume on the high-temperature performance of asphalt, a linear correlation of the temperature increment and rutting resistance factor of the variables was performed. The correlation coefficient between the rutting resistance factor and the temperature increment was very high. The relationship may be described as:

(1) $$G^{*}/\sin\delta ={\mathrm{Ae}}^{\mathrm{Bt}}$$

where t is the increasing temperature range, and A and B are the regression coefficients.

By taking the logarithm of both sides simultaneously, the relationship becomes:

(2) $$\ln\left(G^{*}/\sin\delta \right)=\ln A+\mathrm{Bt}$$

where ln A is the linear intercept, which indicates the initial degree of influence of the silica fume content on G*/sin δ, and B is the slope of the linear function, which represents the rate of decrease of G*/sin δ with the increase in temperature (i.e., the extent of the influence of the silica fume content on G*/sin δ) [36]. The results of the constructed regression equation are shown in Table 5 and Table 6.

In Table 5 and Table 6, it is shown that the correlation coefficient (R^2^) of the asphalt before and after aging was greater than 0.99, indicating that G*/sin δ exhibited a good linear relationship with the temperature in natural logarithmic coordinates [36]. ln A increased with the addition of silica fume, indicating that the rutting resistance factor increased accordingly. Additionally, the initial influence of the modifiers on G*/sin δ also increased, and the improvement of the high-temperature performance of asphalt became more evident. After aging, the asphalt exhibited an improved high-temperature performance owing to the transformation of light components to heavy components, and therefore, the value of ln A was greater than that of the composite-modified asphalt before aging. As the silica fume content was increased, the range of increase of ln A also gradually increased.

Moreover, as the silica fume content was increased, the value of |B| gradually decreased, indicating that the addition of silica fume can reduce the temperature sensitivity of asphalt and improve its high-temperature stability. The increasing range of |B| also gradually decreased after aging, which indicates that aging has a greater impact on the SBS-modified asphalt than on the silica fume/SBS composite-modified asphalt.

### 3.4. Low-Temperature Rheological Properties

**Figure 3:**
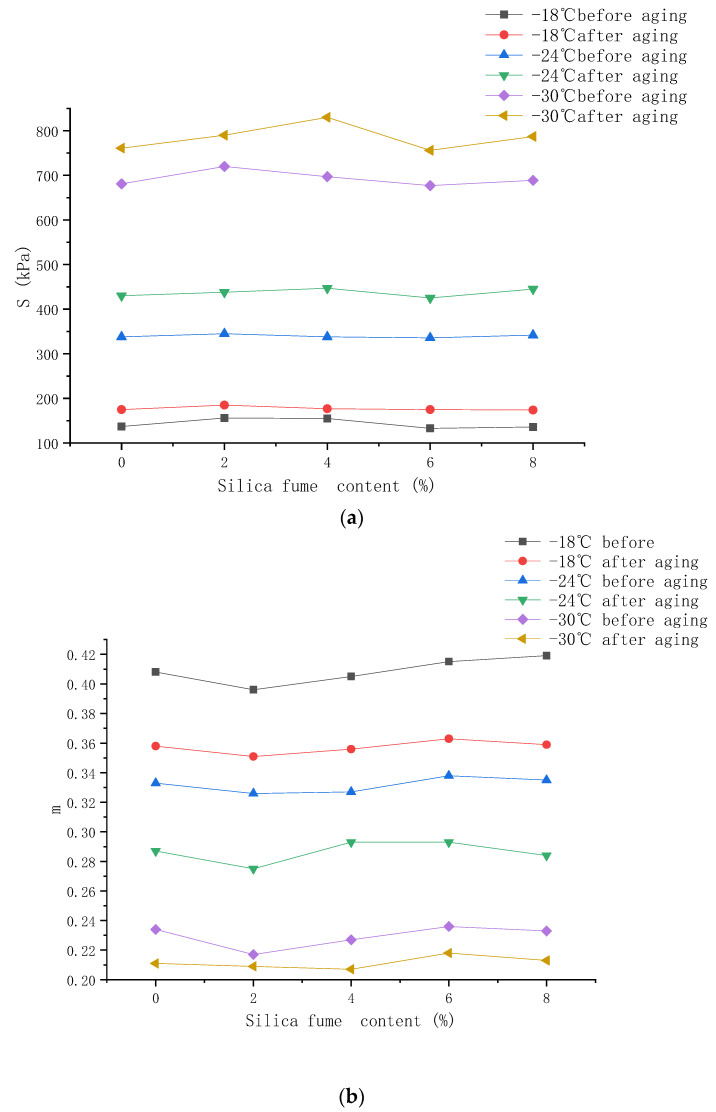
(**a**) Creep stiffness modulus (S) of the asphalt before and after aging; (**b**) creep rate (m) of the asphalt before and after aging.

This section compares the low-temperature rheological properties of 4% SBS-modified asphalt and composite-modified asphalt with 2%, 4%, 6%, and 8% silica fume contents before and after aging through the BBR test. In the low-temperature BBR test, S can be used to characterize the deformation resistance of asphalt at low temperatures. The larger the value of S, the smaller the deformation resistance of the asphalt. m reflects the relaxation ability of asphalt; the higher the value of m, the stronger the low-temperature rheological resistance of the asphalt. The specified value of SHRP is: S < 300 Mpa, m > 0.3. The results of the test are shown in Figure 3.

As shown in Figure 3, both S and m before and after aging do not comply with SHRP regulations when the temperature is lower than −24 °C.

An increase in the amount of silica fume initially led to an increase in the creep stiffness modulus of the composite-modified asphalt at different temperatures, which subsequently decreased. Additionally, under the same conditions, the creep rate initially decreased and then increased. This shows that adding silica fume makes asphalt easier to crack and reduces its low temperature performance [37]. When the silica fume content was 6%, the S value of the composite-modified asphalt was small, and the m value was higher than that of the SBS-modified asphalt. The reason may be that 6% silica fume is a suitable dosage, which can be evenly distributed in asphalt and blended with SBS to form a stable network structure [38].

After undergoing short-term aging, the creep stiffness modulus and the creep rate of the composite-modified asphalt exhibited little variation as the temperature was varied. This shows that although the low temperature performance of the composite-modified asphalt decreases with the incorporation of silica fume, it has no obvious effect on its anti-aging properties. This is because the silica fume and modified asphalt were in a physically miscible state, and no chemical reactions occurred to produce new substances after aging. Although the incorporation of silica fume had a negative effect on the S and m values of SBS-modified asphalt, it still slightly improved the aging resistance of asphalt in a low-temperature environment with a silica fume content of 6%.

### 3.5. Infrared Spectroscopic Analysis

**Figure 4:**
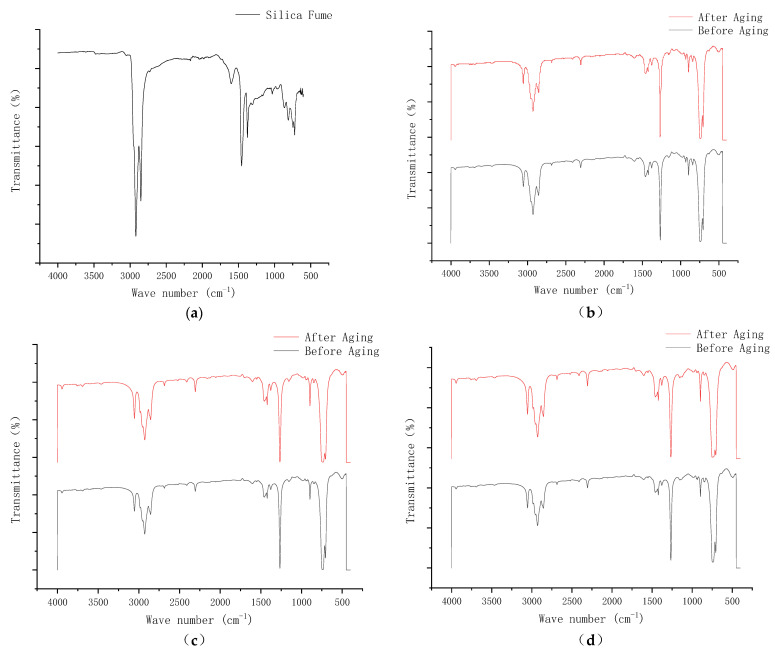

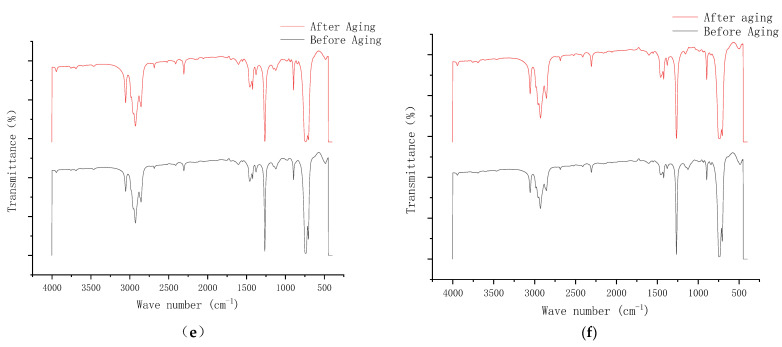
Fourier transform infrared spectra of (**a**) silica fume; (**b**) 4%SBS modified asphalt; (**c**) 4%SBS + 2%silica fume composite-modified asphalt; (**d**) 4% SBS + 4% silica fume composite-modified asphalt; (**e**) 4% SBS+6% silica fume composite-modified asphalt; and (**f**) 4% SBS+8% silica fume composite-modified asphalt.

By analyzing the infrared spectrograms of the composite-modified asphalt samples with different silica fume contents by FTIR spectroscopy, the changes in the functional groups before and after aging of the composite-modified asphalt were observed from a microscale perspective. The infrared spectrograms of the asphalt before and after aging are shown in Figure 4.

#### 3.5.1. Qualitative Analysis

By comparing the spectra that are shown in Figure 4, it is seen that the composite-modified asphalt exhibited the same vibrational absorption peaks as the SBS asphalt, whose peaks almost appeared in the same position. However, the FTIR spectrum of the composite-modified asphalt showed an Si-O stretching vibration absorption peak at 1090 cm^−1^, which was close to the Si-O stretching vibration absorption peak at 1098 cm^−1^ in the silica fume spectrum. This can be attributed to the incorporation of silica fume into the asphalt. In addition, no new absorption peaks appeared in the infrared spectrum of the composite-modified asphalt samples. The characteristic peaks of these samples did not exhibit large displacements, and the absorption peak intensities did not differ significantly. Therefore, it is inferred that no complicated chemical reactions occurred upon the addition of silica fume to the asphalt, no new functional groups were generated, and this addition was simply a physical blending process. This is consistent with other research results [28].

However, by analyzing the infrared spectrum of the asphalt with each silica fume content before and after aging, it was found that the intensities of the absorption peaks of the asphalt mixed with silica fume were reduced near the characteristic peak positions of the asphalt after aging at 1708 cm^−1^ and 1024 cm^−1^ in the aged samples [39]. Therefore, the addition of silica fume suppressed the effects of aging of the asphalt to a certain extent. 

#### 3.5.2. Quantitative Analysis

Thermal oxidative aging breaks the C=C bonds of alkanes and alkyl side chains in the asphalt. The oxidation reaction proceeds under the influence of heat, and a series of oxygen-containing compound carbonyl peaks are formed. As the aging time was extended, the reaction progressed and the carbonyl content increased, which, in turn, led to the growth of the carbonyl absorption peak. The C=C bonds in asphalt reacted with thiols to form thio-ethers, which were further oxidized to form sulfoxide groups. Therefore, the increase in the number of carbon-oxygen double bonds in the carbonyl group and sulfur-oxygen double bonds in the sulfoxide group was used to determine the degree of aging of the asphalt [30]. However, the variation in the sulfur content fluctuates during the short-term aging process, and, therefore, the carbonyl peak area may be used to evaluate the short-term aging of road asphalt [40].

Therefore, we used the carbonyl index (CI) to characterize the aging behavior of the asphalt in this study, which may be defined as:

(3) $$\mathrm{CI}=\frac{A_{C=O}}{A_{C-{\mathrm{CH}}_{3}}}$$

Here, AC=O represents the carbonyl absorption peak area, and AC-CH3 represents the saturated C-H bending vibration absorption peak area.

OMNIC was used to measure the peak area and calculate the CI of the asphalt before and after aging. The results are listed in Table 7.

It can be seen from Table 7 that the CI values of asphalt increased by varying degrees after aging, and that the range of increase in the CI of the composite-modified asphalt was less than that of the SBS-modified asphalt. This finding indicates that adding silica fume to SBS-modified asphalt can effectively inhibit the generation of carbonyl groups during thermal oxidative aging and slow the aging process, thereby improving the aging resistance of asphalt [41,42]. When the silica fume content was 6%, the range of increase in the CI for the composite-modified asphalt after aging was the lowest (48.35%). This indicates that when the silica fume content was 6%, the improvement of the short-term aging of SBS-modified asphalt was the most apparent. This is consistent with the findings of [28,29,35].

## 4. Conclusions

In this study, different amounts of silica fume were used to prepare silica fume/SBS composite-modified asphalt samples that were subjected to short-term aging. According to three-major-indices, DSR, BBR, and FTIR tests, the results of this study are as follows:

1. With the addition of silica fume and the increase of its content, the high temperature durability of composite-modified asphalt before and after aging can be significantly improved. It was observed that the incorporation of silica fume facilitated the retention of the original properties of the modified asphalt and effectively reduced the aging effects on the structure of the modified asphalt.
2. Silica fume will reduce the low temperature rheological properties of composite-modified asphalt. Therefore, in terms of its practical engineering application, silica fume/SBS composite-modified asphalt is recommended to use in an environment where the minimum temperature does not exceed −24 °C. It is necessary to deeply analyze and find ways to improve the low temperature anti-aging performance of composite-modified asphalt.
3. Silicon powder is physically miscible in SBS-modified asphalt; it does not affect the chemical structure of asphalt before and after short-term aging. To inhibit asphalt aging, the generation of carboxyl groups during the thermal oxidative aging process must be reduced; this improves the aging resistance of the asphalt. When the amount of silica fume was 6%, the CI value increased the least among all samples, and this asphalt sample had the strongest short-term aging resistance.
4. Considering the results of predecessors and this paper, it can be determined that the rheology and durability of composite-modified asphalt are the best when the content of silica fume is 6% and the performance of SBS modified asphalt can be significantly improved. Moreover, at this dosage, the cost of asphalt will only increase by $10~$15 per ton. It can greatly improve the performance and durability of the composite-modified asphalt at very low expense. In this way, the pavement performance and service life will be significantly improved.

## Figures and Tables

**Table 1:** Properties of the 4% styrene-butadiene-styrene-modified asphalt.

Penetration (100 g, 5 s, 25 °C) × 0.1/mm	Softening Point (Ring and Ball Method)/°C	Ductility (5 cm/min, 5 °C)/cm
81.1	60.4	35.8

**Table 2:** Silica fume properties.

Color	Mass Fraction of SiO_2_%	PH	Fineness/%	Particle Size/μm	Loss on Ignition	Moisture Content/%	Specific Surface Area/m^2^g^−1^
Gray	≥96	6~8	3	0.1–0.3	≤5	<5	25.37

**Table 3:** Three major indices of the silica fume/ styrene-butadiene-styrene composite-modified asphalt with different silica fume contents before aging.

Silica Fume Content	Penetration (100 g, 5 s, 25 °C)/ 0.1/mm	Softening Point (Ring and Ball Method)/°C	Ductility (5 cm/min, 5 °C)/cm
0%	81.1	60.4	35.8
2%	79.6	61.3	32.9
4%	76.9	63.5	32.3
6%	71.2	66.1	30.8
8%	68.8	65.9	29.3

**Table 4:** Three major indices of the silica fume/ styrene-butadiene-styrene composite-modified asphalt with different silica fume contents after aging.

Silica Fume Content	Penetration (100 g, 5 s, 25 °C)/0.1/mm	Softening Point (Ring and Ball Method)/°C	Ductility (5 cm/min, 5 °C)/cm
0%	49.1	68.7	24.1
2%	48.4	67.4	20.6
4%	48.0	69	18.9
6%	46.1	71.2	17.4
8%	44.8	71.1	15.6

**Table 5:** Regression and correlation coefficients of silica fume/ styrene-butadiene-styrene composite-modified asphalt before aging.

Silica Fume Content	ln A	|B|	R^2^
0%	4.7621	0.0662	0.9977
2%	4.7956	0.0659	0.9973
4%	4.8731	0.0655	0.9989
6%	5.1063	0.0648	0.9993
8%	5.0182	0.0649	0.9957

**Table 6:** Regression and correlation coefficients of silica fume/ styrene-butadiene-styrene composite-modified asphalt after aging.

Silica Fume Content	ln A	|B|	R^2^
0%	5.0463	0.0678	0.9934
2%	5.0827	0.0670	0.9958
4%	5.1676	0.0665	0.9979
6%	5.3544	0.0656	0.9995
8%	5.3541	0.0657	0.9982

**Table 7:** Absorption peak area and carbonyl index of composite-modified asphalt before and after aging.

Silica Fume Content		Absorption Peak Area		CI
		C=O	C-CH3	
0%	Before aging	−0.096	4.196	−0.0229
	After aging	−0.011	3.088	−0.0037 (↑0.0192, 83.84%)
2%	Before aging	−0.139	3.561	−0.0391
	After aging	−0.022	6.277	−0.0035 (↑0.0356, 91.05%)
4%	Before aging	−0.134	3.420	−0.0391
	After aging	−0.036	6.656	−0.0054 (↑0.0337, 86.19%)
6%	Before aging	−0.071	3.909	−0.0182
	After aging	−0.071	7.573	−0.0094 (↑0.0088, 48.35%)
8%	Before aging	−0.128	2.866	−0.048
	After aging	0.042	6.374	0.0066 (↑0.0514, 114.73%)

## Data Availability

The data used to support the findings of this study are included within the article.
